# The Prevalence of Notched Audiograms in a Cross-Sectional Study of 12,055 Railway Workers

**DOI:** 10.1097/AUD.0000000000000129

**Published:** 2015-04-27

**Authors:** Arve Lie, Marit Skogstad, Torstein Seip Johnsen, Bo Engdahl, Kristian Tambs

**Affiliations:** 1 Department of Occupational Medicine and Epidemiology, National Institute of Occupational Health, Oslo, Norway; 2 Norwegian State Railways (NSB) Occupational Health Service, Oslo, Norway; and 3 Norwegian Institute of Public Health, Oslo, Norway.

**Keywords:** Audiometric notch, Diagnosis, NIHL, Noise exposure

## Abstract

**Objective:**

Noise-induced hearing loss (NIHL) is one of the most reported occupational diseases internationally. The occurrence of audiometric notches is emphasized in both American and European guidelines for the diagnosis of NIHL. The aim of this study was to describe the prevalence of notched audiograms among railway personnel with and without noise exposure to better assess the usefulness of such notches in the diagnosis of NIHL.

**Design:**

The most recent audiogram from 1994 to 2011 of a total of 12,055 railway workers, age 20 to 65 years, was obtained from the medical records of the occupational health service of the Norwegian State Railways (NSB). The prevalences of three types of notched audiograms, Coles notch, notch index, and 4 kHz notch, were computed, in relation to age, sex, and occupational noise exposure.

**Results:**

Coles notch in either ear was found in 63% of the male railway maintenance workers, exposed to noise levels of 75 to 90 dB(A), compared with 53% of the non-noise exposed (<70 dB(A)) traffic controllers (*p* < 0.001). The corresponding figures for the 4 kHz notch were 31% versus 21% (*p* < 0.001). For the notch index, 61% of the exposed and 51% of the controls had a notched audiogram (*p* < 0.001). For female workers, the prevalence of audiometric notches was lower, and the differences between noise exposed and nonexposed was smaller than those in men. Increasing age led to an increased prevalence of notches.

**Conclusions:**

Audiometric notches commonly occur among both noise-exposed and those not exposed to noise in railway personnel. The usefulness of audiometric notches in the diagnosis of NIHL is therefore limited.

## Abstract 2

Noise induced hearing loss (NIHL) is one of the most reported occupational diseases internationally. The occurrence of audiometric notches is emphasized in both American and European guidelines for the diagnosis of NIHL. The aim of this study was to describe the prevalence of notched audiograms among railway personnel with and without noise exposure in order to better assess the usefulness of such notches in the diagnosis of NIHL. Our results indicate that audiometric notches commonly occur among both noise-exposed and those not exposed to noise in railway personnel. Thus, we conclude that the usefulness of audiometric notches in the diagnosis of NIHL is limited.

## INTRODUCTION

Noise is one of the most common causes of hearing loss (WHO 2004). Noise-induced hearing loss (NIHL) is one of the most reported occupational diseases internationally (EASHW 2005). In Norway, NIHL accounts for more than 60% of the occupational disorders reported to the Labour Inspection Authority (Samant et al. 2008). Hearing loss, however, is mainly related to aging (Nelson et al. 2005; Dobie 2008; ISO 2013). Hearing loss is more common and more severe in men than in women (ISO 2013). Hereditary conditions also play a major role (Gates et al. 1999; Raynor et al., 2009), and about 40% of the variation in age-induced hearing loss is due to genetics (Kvestad et al. 2012).

In developed countries, hearing loss due to occupational noise exposure is decreasing (Nelson et al. 2005; Dobie 2008). This is probably due to reduced exposure in terms of noise reduction measures and the use of protective equipment. According to ISO 1999, the expected hearing loss due to noise exposure is relatively modest—in the order of 5 dB in the noise sensitive part of the hearing range, that is, 3 to 6 kHz, with an exposure level of 85 dB(A) for more than 40 years (ISO 2013). For comparison, the age-induced hearing loss in 60-year-old men is close to 40 dB and in women of the same age, slightly more than 20 dB (ISO 2013).

It is difficult to differentiate between age-induced hearing loss and NIHL (Rabinowitz 2012). The occurrence of audiometric notches, defined as a hearing loss at 3 to 6 kHz compared with higher and lower frequencies, is emphasized in both American and European guidelines for the diagnosis of NIHL (EASHW 2005; Kirchner et al. 2011), but it is also known that NIHL may exist without the presence of a notch (EASHW 2005; Kirchner et al. 2011). Moreover, audiometric notches may also occur without any previous noise exposure (Nondahl et al. 2009; Osei-Lah & Yeoh 2010). Unilateral audiometric notches seem to be more prevalent than bilateral ones (Wilson & McArdle 2013). There is also some disagreement as to exactly what constitutes an audiometric notch (McBride & Williams 2001; Osei-Lah & Yeoh 2010).

Despite the fact that audiometric notches commonly occur among non-noise-exposed individuals, there are still some who consider a notch as proof of NIHL (Anino et al. 2010; Hsu et al. 2013). This can sometimes lead to exaggerated notions of the prevalence of NIHL and perhaps to the implementation of unnecessarily comprehensive preventive measures. Norwegian railway operations, where two of the authors work as occupational physicians (A.L., T.S.J.), is such an example. Norwegian guidelines state that a hearing threshold ≥25 dB for at least one of the frequencies in the range 3 to 6 kHz or ≥20 dB for all frequencies should basically be viewed as a possible NIHL if there is a noise exposure >80 dB(A). The co-occurrence of an audiometric notch suggests that there is an NIHL (The Norwegian Labour Inspection Authority 2013). The lack of a notch makes the diagnosis NIHL less probable. Many train drivers and conductors have hearing threshold that is consistent with the criteria for NIHL. A number of preventive measures, such as to avoid using the whistle on the platform and the use of hearing protection by shunting of trains, were taken without any effect on the hearing. A recent comparison of the hearing of train drivers and conductors with a reference group of non-noise exposed revealed that the hearing sensitivity in train drivers and conductors did not differ from that of non-noise-exposed reference groups (Lie et al. 2013).

Each country has its own guidelines. Many have pointed out the lack of international guidelines for the diagnosis of NIHL as a problem which also makes it difficult to compare research (Rabinowitz 2012). The aim of this study was to describe the prevalence of notched audiograms among railway personnel with and without noise exposure to better assess the usefulness of such notches in the diagnosis of NIHL.

## METHODS

### Study Group

Three groups of railway employees were chosen: Train drivers and conductors, train and track maintenance workers, and a reference group doing traffic controlling and other types of office work. All these groups have to perform a periodic audiometric test as a part of the mandatory health examination at 1- to 5-year intervals depending on age. All the tests are conducted by the occupational health service (OHS) of the Norwegian State Railways (NSB).

An extensive noise exposure assessment program done by the OHS department has revealed a mean (8-hour Leq) noise exposure of 70 to 85 dBA for the train drivers and conductors, 75 to 90 dBA for the maintenance workers, and <70 dBA for the reference group of traffic controllers. Most of the train drivers, conductors, and traffic controllers are recruited at the age of 20+ years, and because railway employees have very little turnover, age minus 20 years is a good proxy for the exposure time. Because the maintenance workers use hearing protection during the most exposed types of work, the exposure level may be somewhat lower than that indicated by the noise measurements.

The hearing sensitivity of the train drivers and conductors has been found to be approximately the same as for the reference group and not different from that of a national reference population of non-noise-exposed persons (Lie et al. 2013), which recently has been included in the newly revised version of the ISO 1999 (ISO 2013) (Engdahl et al. 2005).

The results for the maintenance workers disclosed a small hearing loss in the 3 to 6 kHz area in the order of 3 to 5 dB in another study not yet published.

### Auditory Examination

Madsen Xeta Otometrics pure-tone audiometric testing using TDH-39P earphone headsets in a soundproof booth at frequencies 0.25, 0.5, 1, 2, 3, 4, 6, and 8 kHz was performed by trained nurses. The testing was done in-line with standard procedures according to the Norwegian Labour Inspection Authority (2013). The audiometers were calibrated every second year according to the requirements of the equipment provider. The most recent audiogram from the medical records for the period of 1994–2011 was chosen for the study.

### Audiometric Notches

We used three types of notch definitions. The Coles notch was defined as hearing thresholds at 3 or 4 or 6 kHz of 10 dB or more compared with that at 1 or 2 kHz and 6 or 8 kHz. The criteria established by Coles et al. have been proven to correlate well with clinical assessments (Rabinowitz et al. 2006).

The Notch index > 0 was defined as a mean hearing threshold of 2, 3, and 4 kHz minus the mean of 1 and 8 kHz > 0. It was used in a study of Rabinowitz to follow the progression of notched audiograms and may be more robust against the diminishing of notches caused by the hearing loss of 8 kHz in presbycusis (Rabinowitz et al. 2006). It is quite similar to what Coles describes as a bulge in the audiogram (Coles et al. 2000).

The 4 kHz notch was defined as a hearing threshold of 4 kHz at least 10 dB greater than that at 2 and 8 kHz and has been regarded as the signature notch in audiograms of NIHL (Wilson 2011).

### Ethical Considerations

According to Norwegian regulations, an application to the regional ethics committee is not necessary because the audiograms have been obtained as a part of regular OHS work where risk assessment of NIHL is an important task.

### Statistics

The data analysis was performed by using SPSS (IBM Statistics version 20). Descriptive statistics were reported. Groups were compared using χ^2^ tests for categorical variables and analysis of variance for continuous variable. For the sex and age adjustments of audiometric findings, the UNIANOVA procedure in SPSS was used, and for the multivariate analysis of categorical variables binary logistic regression was used.

## RESULTS

In the study, we used the most recent audiogram, obtained in the period 1994–2011, for 12,055 railway workers, 9881 men and 2174 women (Table 1). Train and track maintenance workers constitute the largest group. It consists mainly of men and is a little older than the other two groups. This group has higher noise exposure and more hearing loss in the noise sensitive area (3–6 kHz) and also at lower frequencies (0.5 to 4 kHz) compared with the reference group. The group of train drivers and conductors is the youngest one with more women than the group of maintenance workers. Their hearing is approximately equal to the hearing of the reference group. The reference group has the highest percentage of women, 29%.

**Table 1.**
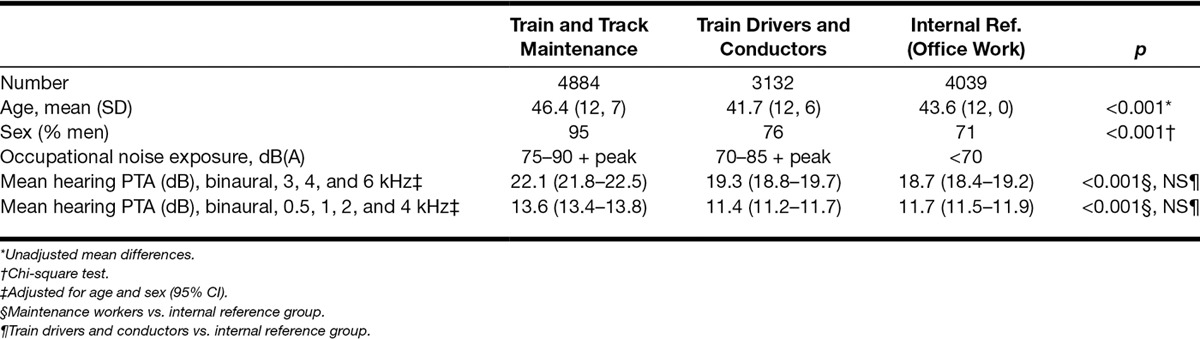
Background data for the three groups of railway workers

**Fig. 1.**
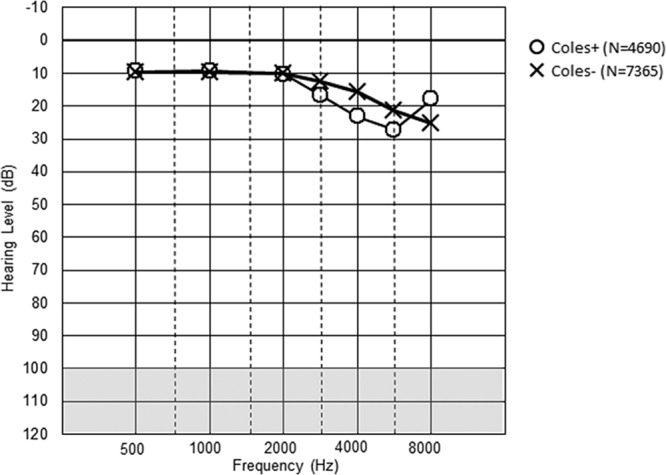
Description of hearing thresholds in Coles notch in the right ear, both sexes (N = 12055). Mean values are given.

**Fig. 2.**
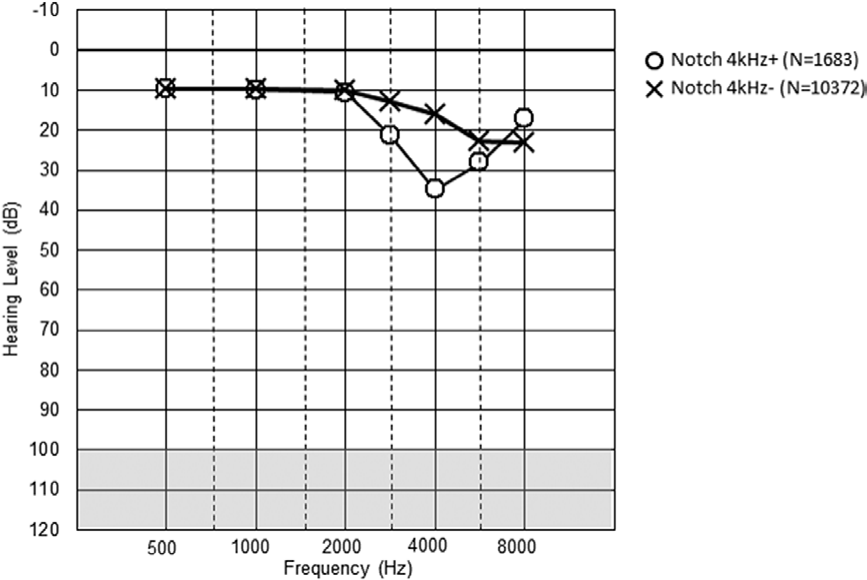
Description of hearing thresholds in various 4 kHz notch in the right ear, both sexes (N = 12055). Mean values are given.

**Fig. 3.**
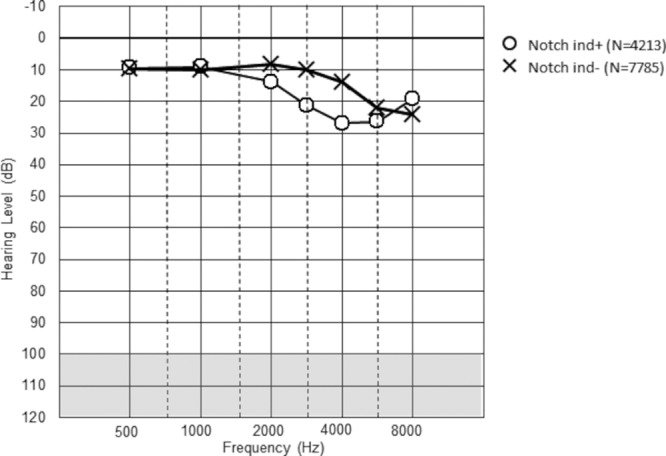
Description of hearing thresholds in Notch index >0 in the right ear, both sexes (N = 12,055). Mean values are given.

Figures 1 to 3 show the mean hearing threshold for the right ear for the three types of notches for all the railway workers. The average depth of the Coles notch was less than 10 dB for the frequencies 3 to 6 kHz. The notch index > 0 was somewhat deeper, whereas the 4 kHz notch was the deepest, close to 20 dB. All the notched audiograms show better hearing at 8 kHz, as expected from the definition of notches. The hearing loss at 2 kHz at notch index > 0 reflects the definition of the notch index.

Table 2 shows the prevalence of notched audiograms in the three occupational groups. The Coles notch, 4 kHz notch, and notch index > 0 occur more often in the group with the highest noise exposure. Although there are significantly more notches of all three types in the highest exposed group, the occurrence of notches is also highly prevalent in the reference group. In either ear, the occurrence of the Coles notch is 63% among the male maintenance workers compared with 53% of the reference group (*p* < 0.001). The 4 kHz notch was found in 31% of male maintenance workers compared with 21% in the reference group (*p* < 0.002), and notch index > 0 was detected in 61% of the male maintenance workers compared with 51% in the reference group (*p* < 0.001). The prevalence of notched audiograms among male train drivers and conductors is in between the prevalences among the maintenance workers and in the reference group. This shows a dose–response relationship between noise exposure and the occurrence of notches, but the results also show that notches are indeed common among employees not exposed to occupational noise.

**Table 2.**
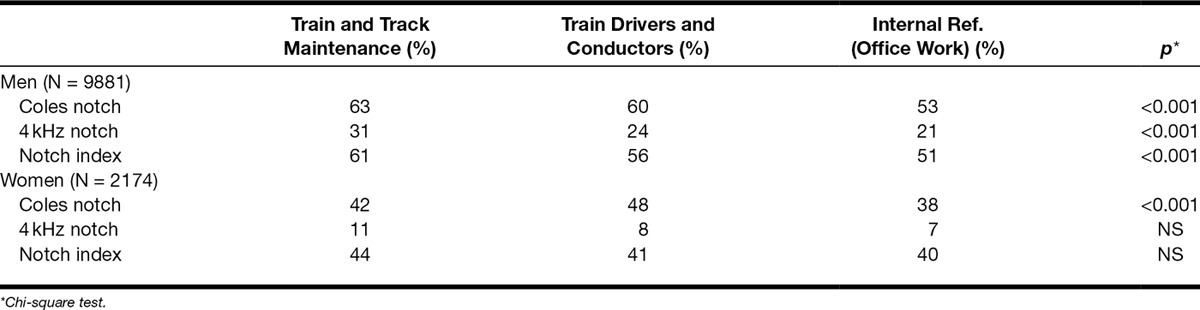
Prevalences of audiometric notches (%) in either ear in relation to occupation and sex

For the female maintenance workers and train drivers and conductors, the prevalences of notches and the differences compared with the reference group were smaller and significant for the Coles notch only.

Unilateral notches are more frequent than bilateral ones (Table 3). The prevalences in men are higher than those in women and tend to increase with age, at least up till around 50 years.

**Table 3.**
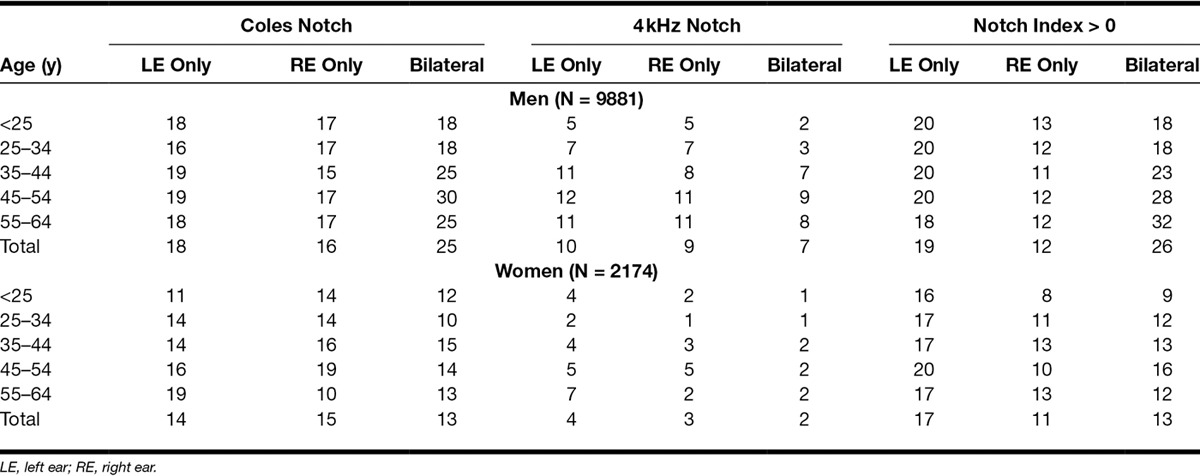
Prevalences of audiometric notches (%) in relation to age, sex, and ear

Because age, sex, and exposure to noise appear to be associated with notched audiograms, we analyzed the data in a logistic regression model (Table 4). The analysis shows that age, noise exposure, and sex are significantly associated with audiometric notches. The importance of age appears to be strongest for the 4 kHz notches. For the Coles notch and the 4 kHz notch, there appears to be a slight decline for the group 55 to 64 years, possibly due to these notches diminishing due to presbycusis, while the notch index is less affected by presbycusis.

**Table 4.**
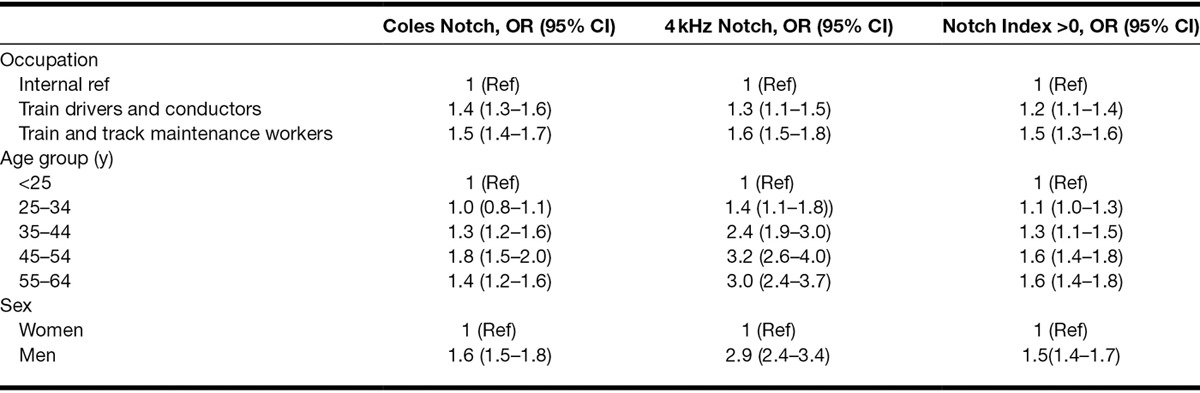
Binary logistic regression of the risk of audiometric notches in either ear associated with noise exposure (occupation), age and sex

Last, we examined the hearing threshold between the three occupational groups adjusted for age and sex in relation to the occurrence of notched audiograms or not. Table 5 shows that the hearing loss of the maintenance workers compared with the reference group is about 1 to 3 dB for both those with and those without notches, suggesting that the noise-related hearing loss is independent of notched audiograms.

**Table 5.**
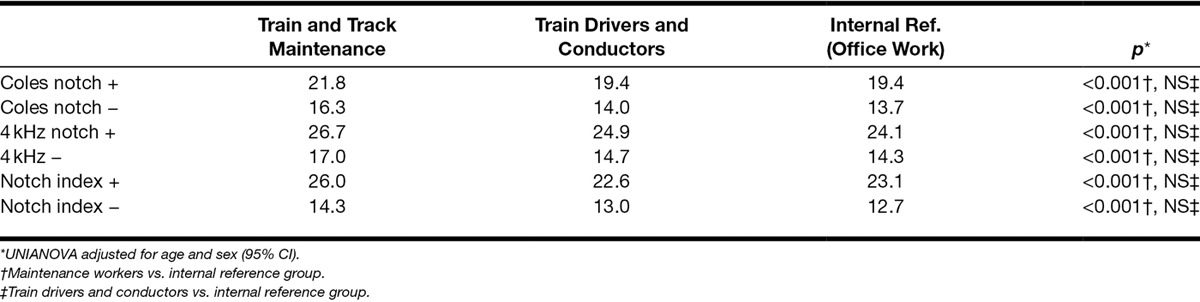
Mean binaural PTA (dB), 3, 4, and 6 kHz, in relation to occupation and audiometric notches adjusted for age and sex

## DISCUSSION

This study shows that notched audiograms are commonly occurring both in workers exposed and not exposed to noise. The prevalence varies with the type of notch. In male workers, Coles notch and notch index > 0 occur in a similar manner, about 60% in noise-exposed and 50% in nonexposed. The prevalence of the 4 kHz notch is lower, 31% in noise-exposed and 21% in nonexposed male workers. Unilateral notches are more common than bilateral. In men, the prevalence of notches increases with increasing noise exposure and age. The prevalence for Coles notch and 4 kHz notch is highest in the age group 45 to 54 years, and then declines. The prevalence of the notch index > 0 continues to increase with age.

In women, it is different. The prevalences of notches is about half of that in men. The relationship with noise exposure is weaker than for men.

The findings of increased prevalences of notches among train drivers and conductors compared with the reference group was surprising because they have a hearing acuity highly similar to the reference group and a noise exposure level that is unlikely to have any significant effect on hearing. We found that train drivers and conductors had slightly worse hearing at 6 kHz (2.1 dB) and a slightly better hearing at 8 kHz (1.4 dB) compared with the controls. Because 8 kHz is central to all definitions of audiometric notches, this small difference probably explains the occurrence of more notches in the train driver and conductor group than in the reference group, despite the small difference in hearing between the groups and the reference group.

This study has some strengths. The number of participants is large. Because a hearing test is compulsory for individuals in all participating groups defined as safety personnel, we believe that the participation rate is close to 100%. To our knowledge, no other studies of this size have looked at the prevalences of notches in relation to age, sex, and noise exposure. The noise exposure data make it possible to provide good estimates of the prevalences of notches at different exposure levels. In addition, we have a large group of nonexposed workers with a hearing acuity comparable with national figures (Engdahl et al. 2005) and international normal values such as ISO 1999 (ISO 2013). However, we lack important information. This includes number of years of employment and other important risk factors for hearing loss, such as use of hearing protection, exposure to leisure noise, smoking, hypertension, and diabetes. We therefore cannot rule out that factors other than railway noise may have influenced the prevalences of notched audiograms.

This study is a cross-sectional study; longitudinal data would be desirable. We cannot rule out selection mechanisms such as those related to health requirements for jobs, including hearing acuity. The health requirements are, however, the same for the three occupational groups, and it is very unusual that someone will leave jobs because of hearing impairment because the health requirements are not very strict. In the jobs under study, one usually starts working at the age of 20 to 30 years and there is little turnover. We therefore believe that the lack of confounder control has not affected the results substantially.

Our results are in accordance with those of Nondahl et al. (2009), who also found that men had Coles notches twice as often as women. Furthermore, in the study by Nondahl et al., occupational noise was associated with slightly higher prevalence of notched audiogram than in our study. The prevalence of notches in the study by Nondahl et al. declined with age, probably because the participants were much older, 58 to 100 years (Nondahl et al. 2009) compared with 20 to 65 years in our study. Taking the age difference into account, the prevalence rates of both Coles notch and Notch index > 0 were compatible in the two studies.

Wilson examined the prevalence of 4 kHz notches in a group of military veterans and found a similar prevalence of notches as in the present group. The prevalence among the veterans for either ear was in excess of 30% in men 40 to 59 years of age compared with 30 to32% in our total material of men 45 to 64 years (Wilson & McArdle 2013). Wilson also found, as in our study, that unilateral notches were more common than bilateral. This is peculiar since noise should have an equal effect on both ears and indicates that noise is only one of several factors that may be responsible for audiometric notches.

Wilson, however, found a slightly lower prevalence of a modified version of Coles notch compared with our study. The 4 kHz notch definition was the same as in our study, but slightly different for the Coles notch that probably explains the higher prevalence in our study (Wilson & McArdle 2013). The study did not have any noise exposure data, but the authors reported that the hearing of military veterans is not different from the civilian counterparts (Wilson et al. 2010).

Various types of audiometric notches are widely prevalent in workers not occupationally exposed to noise and almost as common as in exposed subjects. This makes the diagnosis of NIHL difficult. The prevalence of notches is emphasized in some studies, and some use occurrence of a notch as proof that there is an NIHL (Anino et al. 2010; Hsu et al. 2013). Recently, it has been pointed out that this is a problem (Dobie 2013). This point of view is in-line with several former papers (Clark 2000; Coles et al. 2000; Nondahl et al. 2009; Osei-Lah & Yeoh 2010).

The prevalence of Coles notch and notch index in the present study is approximately 60% in the exposed and 50% in the nonexposed group. This implies that the specificity of one notch as criterion for NIHL diagnosis is very low, as 5 of 6 notches may be unrelated to occupational noise exposure. The general rule that a hearing loss with a notch indicates an NIHL and the lack of a notch speaks against must be used with great caution. The attributable fraction of hearing loss caused by noise at work has decreased in recent years (Nelson et al. 2005; Dobie 2008) in the industrialized part of the world, probably due to reduced noise exposure. This does not make the diagnosis of NIHL any easier.

In conclusion, audiometric notches commonly occur among noise-exposed and non-noise-exposed workers in a Norwegian train company. Age and sex also play a certain role for the prevalence of audiometric notches. The usefulness of audiometric notches as a criterion of the diagnosis of NIHL is therefore of limited value.
